# Ultrasonic strategies for mitigating microbial adhesion and biofilm formation on medical surfaces: a mini review

**DOI:** 10.3389/fmicb.2025.1558035

**Published:** 2025-07-23

**Authors:** Jie Huang, Qiang Fu, Xingang Shao, Yuanzhe Li

**Affiliations:** ^1^College of Polymer Science and Engineering, Sichuan University, Chengdu, China; ^2^Hebei University of Engineering, Shijiazhuang, China; ^3^Carbon Neutrality Institute, China University of Mining and Technology, Xuzhou, China

**Keywords:** biofilm adhesion, ultrasonic techniques, medical surfaces, biofilm disruption, clinical applications

## Abstract

Biofilm formation on medical surfaces poses significant challenges, leading to compromised device functionality and an increased risk of infections. Addressing this issue requires effective strategies that balance efficacy with safety. This mini-review examines the application of ultrasound as a promising approach for biofilm control in medical contexts. Drawing from recent studies, it explores the mechanisms by which ultrasound disrupts biofilms, highlighting its ability to break down extracellular polymeric matrices and enhance the efficacy of antimicrobials. The review also discusses practical considerations, including ultrasound parameter optimization, biocompatibility, and integration with other anti-biofilm strategies. While ultrasound has demonstrated potential in disrupting biofilms, further research is essential to refine these approaches, improve treatment outcomes, and ensure compatibility with medical applications. By advancing our understanding and application of ultrasonic techniques, this field holds promise for improving patient safety and enhancing medical device longevity.

## Graphical Abstract

Schematic representation of ultrasound (US)-induced biofilm disruption. Upon ultrasound wave propagation, the structured biofilm undergoes cellular disruption, leading to detachment and dispersal of biofilm components. This figure was created by the authors. 
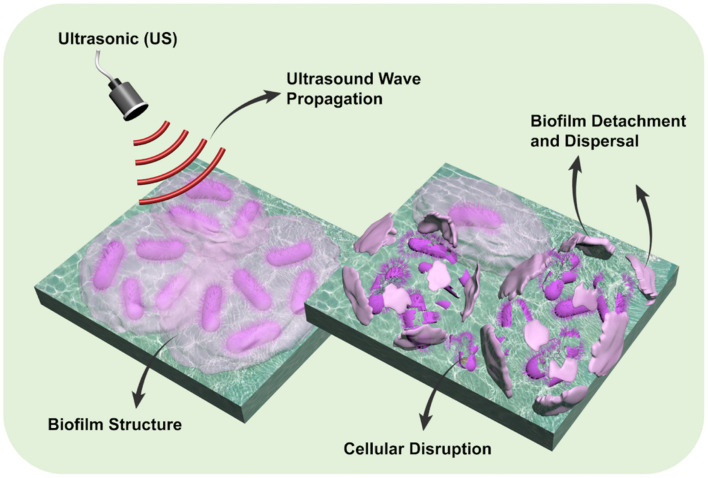

## 1 Introduction

Biofilms, structured colonies of microorganisms enveloped in self-secreted polymeric substances, exhibit an insidious affinity for various surfaces within medical contexts, including indwelling devices like catheters, prosthetics, and surgical instruments, as well as surfaces within healthcare settings. These microbes, embedded within a self-produced matrix, establish microenvironments that safeguard them against external threats while enabling intercellular communication and genetic material exchange. Particularly alarming is the emergence of biofilms hosting pathogenic bacteria, such as *Pseudomonas aeruginosa* and *Staphylococcus aureus*, which are implicated in persistent, often hard-to-treat infection (del Pozo et al., 2009). The entrenchment of these microbial communities on medical surfaces introduces complexities in infection management, often necessitating prolonged and intensified therapeutic interventions, thereby exacerbating healthcare costs and compromising patient outcomes (del Pozo et al., 2009).

The imperative to control biofilm adhesion and proliferation is underscored by the substantial financial and clinical burdens imposed by biofilm-associated infections. In the United States, biofilm-related infections are estimated to cost the healthcare system ~$94 billion annually, with over 500,000 deaths attributed to these infections each year (Cámara et al., 2022). Specifically, waterborne illnesses caused by biofilm-associated pathogens, such as non-tuberculous mycobacteria, Pseudomonas, and Legionella, result in about 7.15 million cases annually, leading to over 600,000 emergency department visits, 118,000 hospitalizations, and 6,630 deaths, incurring direct healthcare costs of ~$3.33 billion (Bryers, 2008; Almatroudi, 2025). Furthermore, chronic wound infections, often associated with biofilm formation, are estimated to cost the U.S. healthcare system over $25 billion each year (Collier et al., 2021). The indelible impact is not just economic but permeates the quality of patient care, often entailing prolonged antibiotic therapies, supplementary surgical interventions, and in certain scenarios, device replacements (Chen et al., 2013). Thus, effective control strategies to inhibit biofilm formation and mitigate their propagation are crucial to attenuating the healthcare burdens and enhancing clinical outcomes.

Ultrasound technology has surfaced as a promising modality for biofilm control, leveraging the physical phenomena of acoustic cavitation to destabilize and disintegrate biofilm structures (Vyas et al., 2019). Acoustic cavitation pertains to the formation, growth, and implosive collapse of microbubbles within a liquid medium, induced by the alternating high-pressure and low-pressure phases of an ultrasonic wave (Paranhos et al., 2007). This collapse generates localized shock waves and high-velocity microjets, capable of disrupting biofilm matrices and bacterial cell walls, thereby offering a mechanical means of biofilm eradication without resorting to antimicrobial agents (Erriu et al., 2014). Yet, the efficacy, specificity, and safety of ultrasound applications in biofilm control, especially *in vivo*, necessitate meticulous scrutiny (Erriu et al., 2014; Vyas et al., 2019). The critical review of current research reveals a divergence in findings, with certain studies corroborating the efficacy of ultrasound in biofilm disruption, while others underscore limitations related to penetration depth, potential impacts on host tissues, and variable efficacy across different microbial species and biofilm maturity stages. This mini-review seeks to elucidate the potential, challenges, and mechanistic underpinnings of utilizing ultrasound in managing biofilm adhesion resistance, providing a consolidated perspective that may guide future research and technological developments in this domain.

## 2 The transition from microbial adhesion to biofilm formation on medical surfaces

### 2.1 The genesis and architectural complexity of biofilms

Biofilms represent a highly organized community of microbial entities, typically bacteria, which adhere to surfaces and generate an extracellular polymeric substance (EPS) matrix (Flemming and Wingender, 2010; Tolker-Nielsen, 2015; van Loosdrecht et al., 1995). The genesis of biofilms commences with the initial attachment of planktonic microorganisms to a substrate, propelled by various factors, including surface properties, microbial adhesins, and environmental conditions (Flemming and Wingender, 2010; Tolker-Nielsen, 2015). Once adhered, these microbes proliferate and commence the synthesis of the EPS matrix, comprising polysaccharides, proteins, nucleic acids, and lipids, which encapsulate the microbial cells, fortifying their adherence and providing a protective milieu (Flemming and Wingender, 2010; Tolker-Nielsen, 2015). The complexity of biofilm architecture is further amplified by the establishment of microenvironments, gradients (nutrient, pH, and oxygen), and inter-microbial interactions, which facilitate a cooperative and often synergistic existence, enabling them to withstand hostile external conditions (Tolker-Nielsen, 2015).

### 2.2 Biofilms: a causative agent for medical complications and material deterioration

Biofilm adhesion to medical surfaces induces a multitude of detrimental consequences, stretching from device failure to severe infections. The EPS matrix serves not only as a physical barrier, shielding microbial cells from antimicrobial agents and host immune responses but also as a medium facilitating horizontal gene transfer among the resident microbes, potentially disseminating antibiotic resistance genes (Madsen et al., 2012; Rux et al., 2023). Biofilm-associated infections pose a significant challenge in medical settings due to their resilience and the substantial economic burden they impose. Recent analyses have highlighted the extensive costs associated with these infections. For instance, in the United States, healthcare-associated infections (HAIs) account for over 88,000 fatalities annually, with an estimated economic burden of USD 4.5 billion (Cámara et al., 2022). Furthermore, a global perspective reveals that biofilms have an economic significance exceeding USD 5 trillion annually, impacting various sectors including healthcare (Mendes et al., 2007; Resch et al., 2006; Zhong et al., 2024a). The formation of biofilms on indwelling medical devices, such as catheters and prosthetics, can lead to device malfunction, material degradation, and persistent systemic infections. These complications often necessitate device removal and replacement, further amplifying healthcare costs. Moreover, biofilm-associated pathogens exhibit enhanced resistance to antibiotics and host defenses, making infections chronic and recurrent (Almatroudi, 2025). Addressing the challenges posed by biofilm-associated infections requires a multifaceted approach, including the development of novel antimicrobial strategies, improved diagnostic tools, and stringent infection control measures. By understanding the economic and clinical implications, healthcare systems can better allocate resources to mitigate the impact of these persistent infections (Mombelli et al., 2018).

### 2.3 Contemporary strategies to mitigate biofilm formation and proliferation

#### 2.3.1 Chemical antimicrobial approaches

Within the domain of chemical antimicrobials, a diverse spectrum of agents, encompassing antibiotics and biocides, has traditionally been employed to counteract the formation of biofilms. The mechanisms by which these agents operate often rely on their ability to penetrate the extracellular polymeric substance (EPS) matrix, disrupt the integrity of microbial cells, and inhibit critical metabolic pathways necessary for the sustenance and proliferation of biofilms (Gilbert et al., 1997; Mah and O'Toole, 2001). However, it is important to recognize that the specter of antimicrobial resistance looms large, as sub-lethal concentrations of these agents within biofilms can potentially foster an environment conducive to the evolution and dissemination of resistance mechanisms (Simões et al., 2009). Moreover, the collateral impacts on nearby biotic entities and abiotic materials underscore the need for judicious utilization and continuous monitoring of these chemical strategies (Jones et al., 2011).

#### 2.3.2 Physical disruption strategies

Physical methods such as ultraviolet light, ultrasound, and mechanical interventions offer effective alternatives for addressing biofilm growth, particularly in cases where chemical resistance is a concern. These strategies provide distinct advantages, especially when applied at appropriate stages of biofilm development. Experimental validation for the efficacy of these biofilm removal methods. Kujundzic et al. (2007) examined the effects of ultrasonic disinfection on *P. aeruginosa* biofilms, demonstrating ultrasound's efficacy in disrupting biofilm structures at early and intermediate stages. Similarly, Qian et al. (1997) investigated the effects of different ultrasound frequencies on biofilm removal efficacy, supporting ultrasound's role in early-stage biofilm disruption. For UV light efficacy, Lim et al. (2022) evaluated the impact of UV-C irradiation on bacterial biofilm reduction, particularly in chronic infections, demonstrating the wavelength-dependent effects of UV exposure. Additionally, Thurnheer et al. (2014) explored the penetration of UV and other antimicrobial agents into biofilm matrices, revealing structural factors affecting treatment efficacy. The role of mechanical scraping in biofilm removal has been extensively studied in the context of dental and medical device hygiene, particularly for surfaces such as dentures, catheters, and endoscopic equipment. Vyas et al. (2019) compared mechanical and chemical biofilm removal methods and emphasized that while mechanical scraping can disrupt the biofilm matrix to some extent, it is often insufficient on its own. Their findings highlighted that mechanical methods alone fail to eliminate deeper or more mature biofilms, especially those with complex microbial communities or in porous materials. Therefore, they recommended that mechanical removal be complemented with chemical disinfectants or ultrasonic treatments to achieve more thorough decontamination and prevent recurrence of infection.

However, the mature biofilm stage poses greater challenges, as its dense and resilient structure reduces the effectiveness of all physical methods (Kujundzic et al., 2007; Qian et al., 1997; Thurnheer et al., 2014). Ultrasound technology, already widely used in medical and industrial applications, proves particularly promising in mitigating biofilm adhesion to medical surfaces. Its targeted action makes it an efficient tool for biofilm control, especially during the initial stages of biofilm formation (Khalid et al., 2019). Despite reduced effectiveness in later stages of our study, ultrasound remains a promising non-invasive technique for biofilm control, particularly in clinical and industrial applications. Further optimization of treatment parameters, such as ultrasound frequency, exposure time, and combination treatments, may enhance biofilm removal efficacy across different maturity stages (Yu et al., 2017). Additionally, the observed variability in treatment outcomes highlights the importance of refining intervention strategies based on biofilm maturity and structure (Kujundzic et al., 2007; Qian et al., 1997). Besides, a clear understanding of the limitations and optimal parameters for each physical strategy remains essential for improving outcomes (Thurnheer et al., 2014).

#### 2.3.3 Multi-modal biofilm control strategies

Effectively managing biofilms—especially in persistent or high-risk settings—often requires more than one approach. Relying on a single method, whether chemical, physical, or biological, frequently falls short due to the structural complexity and adaptive nature of biofilms. As such, there is growing interest in multi-modal strategies that integrate two or more mechanisms of action to enhance overall efficacy. One well-documented example is the use of low-frequency ultrasound (LFU) in combination with antimicrobial agents. The ultrasound waves disrupt the biofilm matrix through cavitation, temporarily increasing permeability and allowing deeper penetration of antibiotics or disinfectants. This approach has shown promising results against biofilms formed by *S. aureus* and *P. aeruginosa* on wound dressings and catheter surfaces (He et al., 2021). Another emerging strategy involves pairing enzymatic treatments with conventional antimicrobials. Enzymes like dispersin B or DNase I degrade extracellular polymeric substances (EPS), which are key to biofilm cohesion and protection. When used prior to or alongside chemical treatments, these enzymes significantly improve biofilm dispersal and microbial killing. For example, a study by Lin et al. (2021) demonstrated that DNase I pre-treatment improved the efficacy of chlorhexidine against dental plaque biofilms by over 40%.

There is also increasing interest in biological–chemical hybrids, such as using probiotic bacteria to disrupt pathogenic biofilms through competitive inhibition or bacteriocin production, followed by mild chemical sanitation. This approach has been explored in food processing environments and urogenital health settings, where it helps restore microbiota balance while reducing reliance on aggressive disinfectants (Yan et al., 2021). Furthermore, recent research into nanoparticle-assisted delivery systems has paved the way for combining physical targeting with controlled chemical release. Silver or zinc oxide nanoparticles can be engineered to respond to pH or biofilm-specific enzymes, releasing antimicrobial agents directly into the biofilm core. These nano-enabled systems have been tested in chronic wound models with promising outcomes (Huang et al., 2022). What all these approaches have in common is their emphasis on synergy: one component weakens the biofilm's defenses while the other executes the kill. However, multimodal strategies must be carefully calibrated. Overlapping toxicity, material compatibility, and unintended microbial shifts are real concerns. Therefore, optimization—whether in a clinical trial or industrial process validation—is crucial before full-scale application.

In practice, multimodal strategies are gaining traction in medical device sterilization, dental plaque control, chronic wound management, and even in water treatment systems, where biofilms compromise safety and performance. As we continue to better understand biofilm biology, these layered approaches may well become the standard, offering a more robust and sustainable path forward in biofilm control.

## 3 Analysis of ultrasound intensity and its mechanism in biofilm disruption

Ultrasound waves, characterized by their high energy, possess the ability to reflect off surfaces, and permeate through various porous structures. It is postulated that the shear forces present within the acoustic boundary layer play a pivotal role in facilitating the detachment of bacteria from surfaces (Iqbal et al., 2013; Vyas et al., 2019). To elucidate the mechanisms underpinning the cleaning effect of ultrasound, meticulously designed experiments have been conducted, utilizing adjustable ultrasonic transducers (Bigelow et al., 2019; Iqbal et al., 2013; Qian et al., 1997, 1996). Furthermore, a monolayer sample of biofilm, simulating the initial 24 h attachment phase, has been employed to mimic early-stage biofilm adhesion, providing insights that are crucial for developing targeted ultrasonic interventions for biofilm disruption (Qian et al., 1996).

### 3.1 Mechanism of action: acoustic disruption of biofilm architecture through ultrasound intensity

Ultrasound intensity, denoted as *I*, is a pivotal parameter in ultrasound applications, both diagnostic and therapeutic, and is defined as the power *P* per unit area *A* through which the ultrasound wave propagates, expressed mathematically as *I* = *P/A* (Vyas et al., 2019). This parameter, typically quantified in watts per square centimeter (W/cm^2^), is integral in optimizing the efficacy and safety of ultrasound applications, considering various spatial and temporal characteristics, such as spatial peak (*I*_*sp*_) and average (*I*_*sa*_) intensity, and temporal peak (*I*_*tp*_) and average (*I*_*ta*_) intensity, as well as spatial-peak temporal-average intensity (*I*_*spta*_) (Vyas et al., 2019).

#### 3.1.1 Cavitation phenomenon

The propagation of ultrasound through media, particularly in the context of biofilm eradication, induces a notable phenomenon known as cavitation (Wu et al., 2013). This process encompasses the formation, growth, and implosive collapse of microbubbles within the liquid medium enveloping the biofilm (Wu et al., 2013). The resultant shockwaves and microjets, emanating from the collapsing bubbles, exert substantial mechanical stress, with the potential to disrupt the structural integrity of the biofilm and perturb the embedded microbial cells (Wu et al., 2013). This cavitation phenomenon is intricately linked to the ultrasound intensity, where a higher intensity often correlates with increased cavitation activity, thereby amplifying the mechanical stress exerted on the biofilm (Wu et al., 2013).

#### 3.1.2 Cellular and matrix disruption

The localized mechanical forces, originating from the cavitation phenomenon, possess the capability to compromise the membranes of the microbial cells, inducing a process known as cellular lysis, which leads to the subsequent release of intracellular contents (Sinisterra, 1992; Wu et al., 2013; Zhong et al., 2024a). Concurrently, the extracellular polymeric substance (EPS) matrix, a critical component ensuring biofilm stability and resilience, becomes susceptible to mechanical degradation (Erriu et al., 2014; Wu et al., 2013). This degradation has the potential to attenuate the protective and adhesive capabilities of the biofilm, thereby diminishing its structural integrity and defensive mechanisms (Erriu et al., 2014; Wu et al., 2013). The degree of cellular and matrix disruption is often directly proportional to the intensity and frequency of the ultrasound applied, necessitating meticulous calibration to ensure efficacy while mitigating potential collateral damage to surrounding tissues (Erriu et al., 2014; Wu et al., 2013).

#### 3.1.3 Biofilm detachment and dispersal

Ultrasound application, particularly at specific intensities and frequencies, can facilitate the detachment of biofilm fragments or individual microbial cells, thereby reducing biofilm thickness and surface coverage (LuTheryn et al., 2020; Yu et al., 2020). However, this detachment, while effective in reducing localized biofilm presence, may pose risks associated with the dissemination of biofilm-derived pathogens or genetic elements within the system (LuTheryn et al., 2020; Yu et al., 2020). Consequently, strategic consideration and thorough understanding of the ultrasound parameters and their impact on biofilm detachment and dispersal are imperative in the ultrasound application. This ensures the effective reduction of biofilm presence while preventing inadvertent propagation of biofilm-related challenges within the system.

Navigating through this parameter space involves a profound understanding of biofilm biology, ultrasound physics, and the complex relationship between them (Vyas et al., 2019). It entails careful modulation of ultrasound frequency and intensity to maximize biofilm disruption while mitigating potential risks such as biofilm dispersal, resistance development, and harm to adjacent tissues or materials (Mah and O'Toole, 2001; Simões et al., 2009).

Furthermore, the potential for synergistic effects with other antimicrobial strategies opens up new avenues for enhancing biofilm eradication, especially in cases of resistance or reduced susceptibility (Mah and O'Toole, 2001; Simões et al., 2009).

Ultimately, the strategic application of ultrasound in biofilm management requires a holistic approach, where parameters are optimized in tandem with a deep comprehension of biofilm intrinsic properties, contextual relevance, and biocompatibility considerations (Vyas et al., 2019). Future research should focus on refining ultrasound applications, unraveling underlying mechanisms, and exploring synergies to create *innovative* strategies that balance efficacy with safety, paving the way for more effective biofilm control in diverse medical settings.

### 3.2 Efficacy of ultrasound across diverse biofilm morphologies

#### 3.2.1 Efficacy across specific biofilm types

Biofilms, inherently complex structures formed by microbial communities, exhibit unique architectures, resistances, and behaviors contingent upon their constituent microbial species. The intricacies of these biofilms, from their matrix composition to their spatial organization, influence their susceptibility to external interventions, including ultrasound. For instance, biofilms formed by *S. aureus* in prosthetic joint infections may possess distinct structural and functional attributes compared to those formed by *P. aeruginosa* in respiratory devices (Chittick et al., 2013). These differences can manifest in varied susceptibilities to ultrasound-mediated disruption. A comprehensive understanding of the efficacy of ultrasound across diverse medically relevant biofilms requires a meticulous, data-driven approach, considering both the biofilm's intrinsic properties and the ultrasound parameters employed.

Ultrasound-based biofilm control has been extensively explored across diverse applications, including prosthetic devices, water treatment systems, and wound care. However, no universal protocol exists, as the success of ultrasonic disruption depends heavily on the specific biofilm composition, maturity, and the material or device surface involved. Ultrasound parameters such as frequency, intensity, and application mode (direct or indirect contact) interact with variables like microbial species, extracellular polymeric substance (EPS) density, and whether the bacteria are in planktonic or biofilm states. These factors make clear that a one-size-fits-all approach is ineffective and underscore the importance of application-specific optimization.

The comparative data across Tables 1, 2 emphasize a crucial distinction in ultrasound-mediated microbial inactivation: the differential response of biofilm-associated and planktonic bacterial populations. These two microbial states differ markedly in structure, resistance mechanisms, and clinical relevance—necessitating distinct treatment strategies.

a) **Ultrasound efficacy on biofilms**
Ultrasound has demonstrated promising efficacy against biofilms, especially those formed by single-species populations. As shown in Table 1, most studies involving *S. aureus, E. coli*, or *P. aeruginosa* report high or moderate-high biofilm disruption at ultrasound frequencies of 20–40 kHz and intensities above 0.5 W/cm^2^. These parameters likely optimize acoustic cavitation, which exerts mechanical shear forces, enhances permeability, and disrupts the extracellular polymeric substance (EPS) matrix. Single-species biofilms, such as those formed by *S. epidermidis* or *MRSA* on orthopedic and implantable surfaces, consistently showed high sensitivity, with up to 90% reduction in biofilm biomass. This is likely due to their simpler structure and lack of interspecies metabolic buffering. In contrast, multi-species biofilms (e.g., *S. aureus* + *P. aeruginosa* in catheter coatings or wound sites) generally exhibited moderate to high efficacy, but required higher intensities or synergistic agents for comparable disruption. These consortia often benefit from cooperative stress resistance and EPS diversity, which attenuate ultrasound penetration. Interestingly, biofilms in chronic wound environments or on medical device coatings were found to be particularly responsive to high-frequency (e.g., 900 kHz) or pulsed ultrasound regimes, such as in LuTheryn et al. (2019). These modalities may offer enhanced delivery of mechanical energy to more mature or hydrated matrices.
b) **Ultrasound efficacy on planktonic bacteria**
Planktonic cultures, analyzed in Table 2, exhibit generally greater susceptibility to ultrasound, with consistent log reductions (>5-log CFU/mL) across multiple strains and contexts. Unlike biofilms, planktonic cells are free-floating and lack protective EPS matrices, making them more vulnerable to cavitation and cell membrane disruption. Recent studies (e.g., Wen et al., 2025) highlight enhanced bactericidal efficacy when ultrasound is applied synergistically with antimicrobial agents (e.g., ε-polylysine or antibiotics). For example, Bai et al. (2024) achieved >5-log inactivation of *S. aureus* in water systems at intensities of 0.5–1.0 W/cm^2^. These results affirm ultrasound's potential as a non-thermal adjunct in disinfection, food safety, and pharmaceutical applications. However, unlike biofilms, ultrasound-induced bacterial death in planktonic systems often depends less on frequency optimization and more on total energy dose and exposure time. Short bursts (e.g., 2–5 min) of low-frequency ultrasound (20–40 kHz) are sufficient to achieve high reductions in most cases.

**Table 1:** Comparative efficacy of ultrasound on biofilms.

**Study (references)**	**Microorganisms tested**	**Application**	**Biofilm type**	**Ultrasound frequency (kHz)**	**Ultrasound intensity (W/cm^2^)**	Efficacy^*^
Nagamanasa et al. (2017)	*S. epidermidis*	Orthopedic implants	Single-species biofilm	20	Not specified	High
Dong et al. (2018)	*S. epidermidis*	Device surface	Single-species biofilm	300	0.5	High
Wang et al. (2018)	*S. aureus*	Implanted medical devices	Single-species biofilm	40	0.09-0.18	High
Zhou et al. (2020)	*K. pneumoniae*	Medical device surfaces	Single-species biofilm	20–40	0.5–1.0	High
Yu et al. (2021)	*MRSA*	Implant surfaces	Single-species biofilm	40	0.3	Moderate-High
Chen et al. (2013)	*P. aeruginosa*	Wound biofilms	Single-species biofilm	40	1.2	High
Yu et al. (2021)	*MRSA*	Implant biofilms	Single-species biofilm	40	0.5	High
He et al. (2021)	*S. epidermidis*	Implant-associated biofilms	Single-species biofilm	20–40	~1.0	High
Lin et al. (2021)	*S. aureus*	Prosthetic joint infections	Single-species biofilm	~20	~1.0	High
Gong et al. (2022)	*S. aureus*	Chronic wound models	Single-species biofilm	~40	~1.0	High
Gopalakrishnan et al. (2022)	*S. aureus, P. aeruginosa*	Polymicrobial biofilms	Multi-species biofilm	20–40	0.5–1.0	High
LuTheryn et al. (2019)	*P. aeruginosa*	Chronic wounds	Single-species biofilm	900	Not specified	High
Huang et al. (2022)	*P. aeruginosa, E. coli*	Wound dressings	Multi-species biofilm	20	1.5	High
Wang et al. (2023)	*Pseudomonas aeruginosa*	Laboratory biofilm models	Single-species biofilm	20–40	0.5–1.0	Moderate-High
Wang et al. (2024)	*S. aureus, P. aeruginosa*	Catheter coatings	Multi-species biofilm	25	2	Moderate-High
Liu et al. (2024)	*Staphylococcus aureus* (MRSA)	Implant-associated infections	Single-species biofilm	20–40	0.5–1.0	High
Lee et al. (2025)	*E. coli*	Dental biofilms	Single-species biofilm	30	0.8	Moderate
	*S. aureus, E. coli*	Indwelling medical devices	Multi-species biofilm	20–40	0.5–2.0	High

^*^Efficacy levels are categorized as follows:

Low: < 40% reduction or removal of biofilm mass or viable cells.

Moderate: 40–70% reduction.

High: 70% removal or inactivation.

**Table 2:** Studies on planktonic cultures (not biofilm).

**Study (references)**	**Microorganisms tested**	**Application**	**Culture type**	**Ultrasound frequency (kHz)**	**Ultrasound intensity (W/cm^2^)**	Efficacy^*^
Scherba et al. (1991)	*E. coli, S. aureus, B. subtilis, P. aeruginosa*	Lab study	Planktonic (individual cultures)	26	0.2–0.5	Moderate
Rediske et al. (1998)	*E. aerogenes, S. marcescens, S. derby, S. mitis, S. epidermidis*	Oral healthcare	Planktonic	70	3	Moderate
Declerck et al. (2010)	*L. pneumophila, A. castellanii*	Water disinfection	Planktonic (cross-kingdom)	36	2–38	Moderate
Yang et al. (2020)	*E. coli*	Surface Sanitization	Planktonic	60	1	Moderate
Bai et al. (2024)	*S. aureus*	Water disinfection	Planktonic	20–40	0.5–1.0	Moderate
	*MRSA, ESBL-producing strains*	Clinical isolates	Planktonic	20–40	0.5–2.0	High
Zhang et al. (2025)	*E. coli, S. aureus*	Pharmaceutical applications	Planktonic	20–40	0.5–1.0	Moderate to High

^*^Efficacy levels are categorized as follows:

Low: < 40% reduction or removal of biofilm mass or viable cells.

Moderate: 40–70% reduction.

High: 70% removal or inactivation.

Table 3 presents a conceptual summary of how variations in ultrasound intensity and frequency affect biofilm disruption and microbial behavior, compiled from multiple published studies, including Vyas et al. (2019), Phull et al. (1997), and Scherba et al. (1991). Unlike Table 1 or Table 2, which is based strictly on empirical outcomes from specific experimental studies, Table 3 is derived from a broader review of the literature to illustrate the general principles governing ultrasound–biofilm interactions across medical and therapeutic contexts. Vyas et al. (2019), although not included in Table 1 due to a lack of discrete species-level biofilm efficacy data, contributed critical mechanistic insights into how ultrasound is applied in clinical settings, particularly in therapeutic, drug delivery, and surgical applications. Their findings underscore that high-intensity, low-frequency ultrasound tends to promote stronger cavitation effects, which can physically disrupt the biofilm matrix and lead to significant bacterial clearance. Conversely, low-intensity, high-frequency ultrasound is associated with enhanced molecular diffusion and drug activation but may lack the mechanical force needed to fully penetrate dense biofilm structures—and in some cases, may even support microbial viability by improving internal nutrient gradients. Although both Phull et al. (1997) and Scherba et al. (1991) investigated planktonic rather than biofilm-forming bacteria, their findings still offer valuable insight into how ultrasound parameters influence microbial inactivation. Lower-frequency ultrasound—typically in the range of 20 to 40 kHz—was shown to generate stronger cavitation effects, making it more effective at broadly disrupting microbial cells in suspension. In contrast, higher frequencies tend to deliver more localized energy, which may be less effective in environments where deeper penetration or widespread mechanical action is needed. These distinctions underscore the importance of tailoring ultrasound settings to the specific microbial context and treatment objective.

**Table 3:** Comparative analysis of ultrasonic parameters in biofilm disruption.

**Ultrasound intensity (W/cm^2^)**	**Ultrasound frequency (kHz)**	**Application & impact**	**Observations (references)**
High (> 5)	High (> 500)	Focused ultrasound, often used for surgical purposes.	Aids in drug penetration and tissue rejuvenation (Vyas et al., 2019).
High (> 5)	Low (< 500)	Therapeutic ultrasonic cleaning and hard tissue surgeries.	Strong bacterial disturbance effects due to cavitation (Vyas et al., 2019).
Low (< 3)	High (> 500)	Specialized drug delivery techniques.	Enhances drug activity but may unintentionally support bacterial growth in some cases (Phull et al., 1997).
Low (< 3)	Low (< 500)	Deeper diffusion applications due to extended wavelengths.	Improves intra-biofilm nutrient and oxygen distribution but can promote biofilm growth (Scherba et al., 1991).

In summary, Tables 1, 2 offer a complementary view of both the practical outcomes and underlying mechanisms involved in ultrasound-based microbial inactivation. Table 1 focuses on the empirical effects of ultrasound on biofilms, revealing how different bacterial species and clinical settings respond to variations in frequency and intensity. In contrast, Table 2 centers on planktonic models, providing a clearer picture of why specific ultrasound regimes—particularly in the 20–40 kHz range with moderate intensity—are more effective for dislodging or inactivating free-floating cells. This distinction is crucial, as the success of ultrasound treatment is shaped by a complex interplay of physical and biological variables, including microbial growth state, cavitation intensity, acoustic energy distribution, and local tissue or surface context. While planktonic studies help isolate direct mechanical effects, biofilm-focused data reveal the added challenges posed by extracellular matrices and microbial cooperation. As a result, the optimization of ultrasound protocols must be tailored not only to maximize efficacy in disrupting bacterial communities but also to minimize collateral effects on surrounding tissues or delicate device surfaces—especially in clinical environments where precision and safety are paramount (Scherba et al., 1991; Vyas et al., 2019).

#### 3.2.2 Synergistic approaches in ultrasound applications

Combining ultrasound with other anti-biofilm strategies has emerged as a promising approach for enhancing treatment efficacy. Rather than relying on a single modality, synergistic methods leverage the complementary strengths of physical, chemical, or biological interventions while compensating for their individual limitations. Notably, ultrasound has been shown to significantly improve the effectiveness of antimicrobial agents by disrupting the biofilm matrix, allowing for deeper penetration of therapeutic compounds (He et al., 2021; Lin et al., 2021; Yan et al., 2021). For instance, antimicrobial peptides and enzymes—such as DNase or dispersin B—can be combined with low-frequency ultrasound to mechanically weaken biofilm structures while simultaneously degrading extracellular polymeric substances. This dual-action mechanism not only facilitates more uniform drug delivery but also reduces the required dosage, potentially minimizing cytotoxicity and resistance development (Dong et al., 2018). Studies have also demonstrated that this synergistic effect can vary depending on the bacterial species involved, biofilm maturity, and the physical properties of the infected surface. Several peer-reviewed studies have illustrated these outcomes. Lin et al. (2021) reported that low-frequency ultrasound paired with chlorhexidine enhanced biofilm eradication in *S. mutans*, while Yan et al. (2021) showed improved treatment of *P. aeruginosa* when ultrasound was combined with conventional antibiotics. These findings suggest that ultrasound's ability to transiently disrupt biofilm integrity can amplify the bactericidal effects of chemical agents, particularly in mature or drug-resistant biofilms.

**Figure 1:**
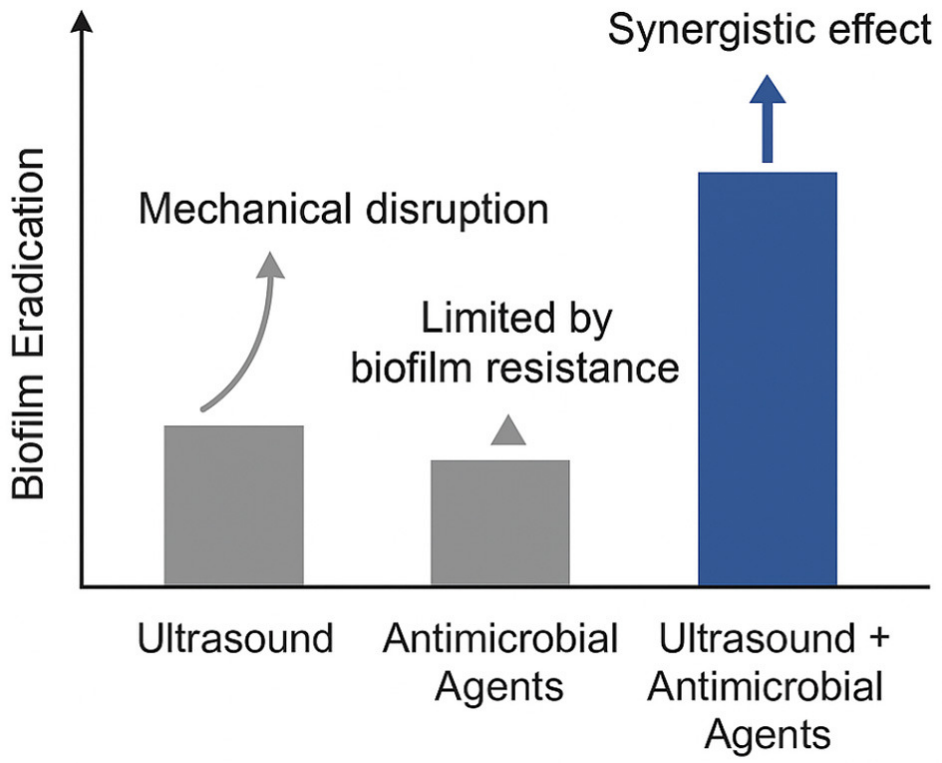
Conceptual illustration of synergistic biofilm disruption via ultrasound-enhanced antimicrobial delivery.

Figure 1 presents a conceptual overview of the enhanced efficacy achieved when ultrasound is combined with antimicrobial agents for biofilm eradication. This schematic is based on findings from multiple peer-reviewed studies that have explored the synergistic potential of mechanical disruption by ultrasound and the biochemical targeting of microbial cells by antimicrobial agents (Lin et al., 2021; Dong et al., 2018). Rather than depicting precise experimental values, the figure summarizes consistent trends reported in the literature: ultrasound alone exerts moderate mechanical effects on biofilms; antimicrobial agents alone often face limitations due to the protective extracellular matrix; but when used together, these strategies yield superior outcomes through improved penetration and bacterial inactivation.

This synergistic relationship has been observed across various bacterial species, including *S. aureus, E. coli*, and *P. aeruginosa*. Ultrasound induces cavitation and microstreaming effects, which transiently disrupt the biofilm matrix and expose embedded bacteria to antimicrobial agents. The result is not merely additive but synergistic, where the combination significantly outperforms either approach used independently. Recent studies provide strong evidence supporting this conceptual model. Zhou et al. (2020) demonstrated that low-frequency ultrasound in combination with antibiotics markedly enhanced biofilm disruption in *Klebsiella pneumoniae*. Similarly, Gong et al. (2022) reported that ultrasound-triggered nanocarriers facilitated deeper and more uniform antibiotic delivery within biofilms. Liu et al. (2024) employed ultrasound-mediated microbubble delivery of vancomycin to effectively target *S. aureus* biofilms, reinforcing the value of acoustic cavitation in overcoming drug resistance.

Additional support comes from Wang et al. (2023), who quantified improved penetration depth of antimicrobial agents facilitated by ultrasound, and Gopalakrishnan et al. (2022), who demonstrated that ultrasound-assisted antibiotic treatment significantly disrupted polymicrobial biofilms, which are typically more resistant to conventional therapies. These studies collectively highlight that ultrasonic enhancement is not limited to one strain or application, but rather offers a versatile adjunctive method in clinical, dental, and industrial contexts where biofilm persistence is a critical challenge.

#### 3.2.3 Balancing efficacy and biocompatibility in ultrasound applications

In ultrasound-mediated biofilm eradication, it is critical to ensure that optimized parameters effectively disrupt biofilms without causing unintended harm to medical devices or host tissues. Achieving this balance requires careful consideration of both efficacy and biocompatibility, especially in medical contexts where safety and functionality are paramount.

The data in Table 4 highlight the variability in ultrasound biocompatibility depending on the application. For example, low-intensity ultrasound used in prosthetic or dental devices demonstrates minimal risks to device integrity and tissue safety. However, higher intensities, such as those in wound care, require greater caution to ensure safety. Each application underscores the need for precise calibration of ultrasound parameters to align with specific use cases. Future research should focus on refining ultrasound techniques to further balance efficacy with biocompatibility. Key areas for exploration include:

- **Mechanistic studies**: Investigating the physical and biological interactions between ultrasound and biofilms.
- **Biofilm heterogeneity**: Understanding how variations in biofilm structure and composition influence treatment outcomes.
- **Combinatorial strategies**: Exploring the integration of ultrasound with antimicrobial agents or surface modifications to enhance biofilm disruption while maintaining safety.

**Table 4:** Overview of ultrasound biocompatibility across various medical applications.

**Medical application**	**Ultrasound frequency (kHz)**	**Ultrasound intensity (W/cm^2^)**	**Device integrity**	**Tissue safety**	**References**
Prosthetic devices / Respiratory devices^*^	26 / 67	0.2–0.5 / 0.3	No erosion / Slight erosion	No tissue damage / Safe	Pitt et al., 1994; Scherba et al., 1991
Dental devices	20–850	0.2–0.07	Maintained	Marginal irritation	Joyce et al., 2003
Cardiac devices^*^	70	Up to 2	Minimal wear	Safe	Pitt and Ross, 2003
Orthopedic implants	28.5	0.5	No erosion	Variable (dependent on strain)	Carmen et al., 2005
Wound care	70	0.5–5	Maintained	Safe under controlled application	Runyan et al., 2006
Implanted medical devices	20 / 300 / 40	0.5 / 0.09–0.18	No structural compromise	High	Dong et al., 2018; Granick et al., 2017; Wang et al., 2018

^*^Suggested field of application according to the article's authors or inferred based on discussion. Experimental confirmation in the specific context may be limited or model-based.

### 3.3. Applications: acoustic interventions across diverse biofilm challenges

#### 3.3.1 Medical device decontamination

Biofilm formation on medical devices, such as catheters, endoscopes, and surgical instruments, poses significant challenges in healthcare settings. Biofilms, such as those formed by *E. coli, S. aureus*, and *P. aeruginosa*, exhibit distinct resistance mechanisms and adherence properties, posing varied challenges in medical device decontamination. *E. coli* biofilms, for instance, are notorious for their resilience and ability to form on a multitude of surfaces, including medical devices, leading to potential healthcare-associated infections (Bouhrour et al., 2024; Elfadadny et al., 2024). Ultrasound has emerged as a promising tool in disrupting biofilms on medical devices. The mechanical effects of ultrasound, particularly cavitation, can disrupt the extracellular polymeric substance (EPS) matrix of biofilms, enhancing the efficacy of antimicrobial agents. For instance, LuTheryn et al. (2020) demonstrated that low-frequency ultrasound significantly reduced biofilm biomass on catheter surfaces, improving subsequent antibiotic penetration.

However, the efficacy of ultrasound in biofilm disruption is influenced by several factors, including biofilm maturity, composition, and the specific ultrasound parameters employed. Studies have shown that while ultrasound can effectively disrupt early-stage biofilms, mature biofilms may require combined approaches. Wang et al. (2023) highlighted the enhanced biofilm eradication achieved by integrating ultrasound with antimicrobial agents, emphasizing the importance of multimodal strategies. Moreover, the heterogeneity of biofilm structures and the potential for residual biofilm fragments post-treatment underscore the need for optimized ultrasound parameters tailored to specific clinical scenarios. Further research is essential to establish standardized protocols that maximize biofilm disruption while ensuring the safety and integrity of medical devices.

#### 3.3.2 Wound management

Chronic wounds often harbor biofilms formed by pathogens such as *P. aeruginosa* and *S. aureus*, which impede healing by resisting host defenses and antimicrobial treatments. *P. aeruginosa* biofilms, characterized by their mucoid phenotype, exhibit enhanced resistance to antibiotics and immune responses, frequently leading to persistent infections. Similarly, *S. aureus* biofilms contribute to delayed wound healing due to their robust extracellular matrices and ability to evade immune detection. Low-frequency ultrasound (LFU) therapy has emerged as a promising adjunctive treatment for disrupting biofilm structures in chronic wounds. The mechanical effects of LFU, particularly cavitation, can disrupt the EPS matrix of biofilms, enhancing the efficacy of antimicrobial agents. For instance, Kvich et al. (2022) demonstrated that combining LFU with antibiotics significantly increased the bactericidal effect against *P. aeruginosa* and *S. aureus* biofilms, achieving up to 99% reduction in viable cells.

However, the application of ultrasound must be carefully managed, as the mechanical forces generated can potentially disseminate biofilm fragments and microbial cells, posing risks of infection spread. Strategies to mitigate this include combining ultrasound with containment or neutralization approaches to manage potential biofilm dispersal. Moreover, the integration of ultrasound with antimicrobial agents has shown synergistic effects, enhancing biofilm disruption while minimizing the risk of dissemination.

Recent studies have also explored the use of ultrasound-responsive microbubbles and nanocarriers to enhance drug delivery and biofilm disruption in wound management. These innovative approaches aim to improve the penetration of antimicrobial agents into biofilms, thereby increasing their efficacy. For example, Lattwein et al. (2022) investigated the use of ultrasound-activated microbubbles to disperse and sonoporate biofilm-associated bacteria, facilitating targeted antibiotic delivery. Ongoing research and clinical trials are essential to optimize these therapies and establish standardized protocols for their effective implementation in wound care.

#### 3.3.3 Dental biofilm control

Dental biofilms, particularly those formed by *Streptococcus mutans (S. epidermidis)* and *Porphyromonas gingivalis* (*P. gingivalis*), play a central role in the etiology of oral diseases such as dental caries and periodontitis (Bowen and Koo, 2018). These species synthesize extracellular polysaccharides that enhance microbial adhesion and biofilm maturation. Traditional ultrasonic scalers, operating typically in the 20–40 kHz range, are routinely applied in dental practice to disrupt these biofilms through cavitation and mechanical vibration (Rediske et al., 1998). However, *in vivo* dental biofilms are rarely monospecies. Clinical biofilms are complex, heterogeneous structures composed of diverse microbial communities embedded in dense extracellular matrices, often exceeding 300 μm in thickness and exhibiting enhanced resistance to both mechanical and chemical interventions. This complexity necessitates evaluating ultrasound efficacy in more representative, multi-species *in vivo* models.

A recent study by Rux et al. (2023) addressed this gap by investigating low-frequency ultrasound (25 kHz) for biofilm removal in extracted human teeth previously colonized *in vivo*. Their optimized protocol achieved up to 79.2% bacterial detachment across polymicrobial biofilms, compared to only 42.6% with conventional ultrasonic debridement without parameter tuning. The study employed quantitative CFU assays and fluorescence-based imaging to confirm these findings, demonstrating that increased amplitude (80 μm) and longer exposure durations (30 s) significantly enhanced biofilm removal without damaging the enamel or dentin surface. These results underscore the importance of tuning ultrasonic parameters—particularly amplitude and duration—for maximum efficacy against real-world biofilms. Furthermore, combining ultrasonic therapy with antimicrobial irrigation (e.g., chlorhexidine or ozone) may amplify efficacy, especially against biofilm niches shielded by structural heterogeneity. Future investigations should also address the risk of bacterial dissemination post-detachment and consider adjunct containment strategies. To conclude, while ultrasound remains a cornerstone of dental biofilm management, its clinical potential can be significantly improved by targeting multi-species, mature biofilms with customized low-frequency protocols. These enhancements offer promising avenues to increase treatment efficacy while maintaining biocompatibility and patient safety (Joyce et al., 2003; Rux et al., 2023).

#### 3.3.4 Medical hygiene pipeline decontamination

Biofilms in medical hygiene pipelines, particularly those formed by *P. aeruginosa* and *L. pneumophila*, pose significant risks by contaminating water systems and compromising sterility in healthcare settings (Declerck et al., 2010). Ultrasound, through acoustic cavitation, generates shear forces capable of disrupting biofilm matrices and mitigating bacterial adherence (Piyasena et al., 2003). However, biofilm resilience and the potential for ultrasound to facilitate biofilm dispersal within pipelines necessitate integrative strategies, such as combining ultrasound with antimicrobial agents to prevent recolonization (Dong et al., 2018). Ensuring the integrity of pipeline materials and compatibility with medical environments is critical to the successful application of ultrasound in pipeline decontamination (Mathieu et al., 2019).

#### 3.3.5 Diagnostic and analytical use of ultrasound in biomaterial-associated infections

While ultrasound is widely recognized for its therapeutic applications in disrupting biofilms, its role in diagnosing and monitoring biomaterial-associated infections (BAIs) has become increasingly important. These infections, often linked to orthopedic implants, catheters, and cardiac devices, tend to be difficult to detect early on due to their slow development and the protective nature of biofilms. Traditional diagnostic methods may fall short, especially when infections are low-grade or located deep within tissues. In this context, ultrasound imaging—particularly techniques like contrast-enhanced ultrasound (CEUS) and elastography—provides a valuable, non-invasive way to assess infection (Li et al., 2020). CEUS, for example, can help visualize abnormal tissue activity around an implant, such as fluid buildup or reduced blood flow—both of which may signal a biofilm-associated infection. A recent clinical study found CEUS to be quite effective, achieving 84% sensitivity and 91% specificity in identifying periprosthetic joint infections, when compared with microbiological culture results (Li et al., 2020).

Ultrasound can also be used to guide aspiration procedures, allowing clinicians to safely extract peri-implant fluid for laboratory analysis. This is particularly useful for identifying the microbial species involved and their antibiotic sensitivities, without the need for surgical intervention. Beyond diagnosis, ultrasound is showing promise in tracking the progress of treatment. For example, clinicians can monitor changes in inflammation or lesion size following ultrasound-assisted debridement or antibiotic therapy. Such real-time feedback can help personalize the course of treatment and avoid under- or overtreatment, which is especially important in long-term infections. That said, ultrasound does have its limitations—for instance, it can be less effective in detecting infections near deep or complex implants, and it may not always distinguish between sterile inflammation and active infection. Even so, its ability to combine diagnostic insight with therapeutic guidance makes ultrasound a versatile tool in managing BAIs. Looking ahead, innovations such as targeted microbubble contrast agents could further improve ultrasound's accuracy in pinpointing biofilm-related infections and assessing how they respond to treatment (Zhong et al., 2024a).

## 4 Unlocking the power of ultrasound: overcoming biofilm challenges

### 4.1 Technical difficulties in ultrasound application

Ultrasound holds great promise as a tool for biofilm eradication, but its practical use faces several technical challenges that need to be addressed (Table 5). One of the most critical aspects is the precision in calibrating and maintaining ultrasonic equipment. The effectiveness of ultrasound depends heavily on accurate settings and routine maintenance of the devices. Without proper calibration, even advanced equipment may fail to achieve consistent and reliable results. Another challenge is the issue of mechanical drift in transducers. Over time, wear and tear can cause deviations in the device's performance, leading to inconsistent ultrasonic wave emissions. This inconsistency can reduce the reliability of the biofilm eradication process, as the delivered waves may vary in intensity or frequency. Additionally, the medium through which ultrasound waves travel introduces variability. Factors such as density and viscosity of the medium can influence wave propagation, potentially altering the impact on the biofilm. If these properties are not optimized, the ultrasound may not penetrate effectively or may affect surrounding structures unintentionally. The overarching challenge lies in ensuring that the ultrasound reaches the biofilm with the appropriate intensity, depth, and consistency while minimizing unintended effects on adjacent tissues or materials.

**Table 5:** Technical challenges in ultrasound application.

**Challenge**	**Sub-challenges**	**Description**	**Implication on biofilm eradication**	**Potential solutions**
Device calibration	- Precision in settings - Regular maintenance	Ensuring devices operate at their optimal settings and undergo routine maintenance.	An improperly calibrated device might not effectively target biofilms.	Implementing standardized calibration protocols.
Mechanical drift in transducer	- Ageing of device - Wear and tear	Over time, the consistency of ultrasonic emissions from transducers can deviate.	Inconsistent wave delivery can lead to unreliable eradication results.	Regular device inspections and timely replacements.
Medium variance	- Density factors - Viscosity elements	Ultrasonic waves can behave differently depending on the medium's density and viscosity.	Variations can affect the depth and strength of wave propagation.	Research to determine optimal mediums for specific biofilm types.

### 4.2 Biofilm resilience to ultrasound

Ultrasound presents a promising avenue for biofilm disruption, but its effectiveness can be hindered by various factors intrinsic to biofilms themselves. Biofilms, structured communities of microorganisms encapsulated within a self-produced matrix, exhibit a variety of characteristics that contribute to their resilience against external forces, including ultrasonic waves. This resilience can be attributed to their physical characteristics, the evolving properties they acquire as they mature, and their strategic positioning within hosts. The subsequent table provides an analytical breakdown of these resilience factors, describing their nature, the mechanism by which they confer resistance to ultrasound, and key considerations for optimizing ultrasound application in light of these challenges (Li et al., 2021). Table 6 dives into the various resilience factors of biofilms against ultrasound and the considerations for applying ultrasound effectively, while providing an objective description for each factor.

**Table 6:** Biofilm resilience factors and their impact on ultrasonic disruptions.

**Resilience factor**	**Objective description**	**Mechanism of resistance**	**Considerations for ultrasound application**
Physical characteristics	Specific traits like thickness, density, and matrix components in biofilms that vary based on their growth and environment.	Biofilms with particular physical traits might be less susceptible to ultrasound waves.	Adjusting ultrasonic parameters, such as frequency and intensity, to match specific physical characteristics can improve the effectiveness of the treatment.
Mature biofilm properties	Over time, biofilms develop viscoelastic properties that can change the way they interact with external forces.	The viscoelastic nature can act like a cushion, mitigating the mechanical effects of ultrasound.	Understanding the maturity stage of the biofilm can guide the customization of ultrasound settings for optimal disruption.
Biofilm positioning	The location of the biofilm, especially if it is situated in intricate or less accessible anatomical sites.	Biofilms in hard-to-reach areas might not receive the full intensity of ultrasonic waves, reducing the efficacy of the treatment.	Employing ultrasound imaging or other modalities to accurately locate and target biofilms can enhance the success rate of the eradication process.

### 4.3 Safety measures and ultrasound protocols

The use of ultrasound for biofilm disruption requires careful consideration of safety measures to prevent unintended harm to tissues, particularly sensitive or delicate ones. Incorrect application of ultrasound can lead to overheating, tissue damage, or cavitation-related injuries. To ensure both efficacy and safety, strict protocols must be followed, and healthcare providers need appropriate training in the use of ultrasonic devices, which is summarized in Table 7.

**Table 7:** Safety considerations in ultrasound application for biofilm disruption.

**Aspect**	**Description**	**Potential consequence**	**Proposed mitigation strategy**
Tissue integrity	Proper application is vital to ensure tissues, especially sensitive ones, aren't inadvertently damaged by ultrasound.	Damage to tissues and complications post-treatment.	Continuous monitoring during procedure and adjusting parameters as necessary.
Risk of overheating	Prolonged exposure to ultrasound can result in tissue overheating.	Thermal injuries to tissues, potentially impairing their function.	Incorporating temperature sensors and auto-cutoff mechanisms in ultrasound devices.
Cavitation hazards	Ultrasound can cause cavitation, leading to potential tissue injuries.	Injuries and tissue damage because of cavitation.	Implementation of cavitation detectors and the selection of appropriate ultrasound frequency and intensity to minimize cavitation risks.
Adherence to protocols	Using the correct frequency, intensity, and duration is paramount for effective biofilm eradication without collateral damage.	Inconsistent results in biofilm disruption and elevated risks of unintended injuries.	Development and rigorous enforcement of standardized operating procedures (SOPs) for ultrasonic treatments.
Provider training	Healthcare providers must possess expertise in the nuanced operation of ultrasonic devices for therapeutic purposes.	Inadequate biofilm eradication and increased chances of procedural complications.	Establishment of comprehensive training programs and certifications for practitioners intending to use ultrasonic methods in treatment.

This includes applying the correct frequency, intensity, and duration tailored to the specific treatment needs. Developing standardized operating procedures (SOPs), conducting regular audits, and incorporating safety features into ultrasound equipment are essential to minimize risks. Additionally, comprehensive training programs for healthcare professionals can enhance their ability to safely and effectively use ultrasonic methods (Zhong et al., 2024b).

Implementing these safety measures and protocols ensures that ultrasound-based treatments achieve their intended biofilm disruption goals while safeguarding patient health. Rigorous SOPs, enhanced device features, and ongoing education for healthcare providers form the foundation of a safe and effective therapeutic approach.

### 4.4 Need for standardized protocols in ultrasound-based biofilm control

While the benefits of ultrasound in biofilm management have been well-documented across a wide range of studies, there remains a significant and unresolved issue: the lack of standardized protocols. Currently, research in this area is highly fragmented. Different studies employ varying ultrasound frequencies, intensities, exposure times, and application methods—each optimized for specific contexts such as dental hygiene, wound care, or orthopedic implants. As a result, drawing direct comparisons between findings becomes difficult, and more importantly, clinicians and engineers are left without clear guidelines for practical, safe, and repeatable use in real-world settings (Li et al., 2020). For instance, some studies report effective bacterial detachment using low-frequency ultrasound (20–40 kHz) at moderate intensities, while others apply higher frequencies or combine ultrasound with chemical agents to achieve desirable outcomes. Yet, these approaches are often not directly transferable between applications due to differences in biofilm maturity, microbial composition, device surface properties, and even anatomical location (Zhong et al., 2024b). Without a harmonized framework, the translation of experimental results into clinical or industrial protocols remains limited.

Developing standardized ultrasonic treatment protocols would not only improve reproducibility but also help ensure patient safety and device integrity. Such protocols could define recommended frequency and power ranges tailored to different infection sites and biofilm types, establish criteria for treatment duration, and set thresholds to avoid unintended tissue or material damage. Furthermore, having well-defined benchmarks would facilitate regulatory approval, equipment certification, and clinician training—crucial steps in moving ultrasound from an experimental solution to a trusted clinical tool (Li et al., 2021). Moving forward, collaboration across disciplines—bringing together microbiologists, biomedical engineers, clinicians, and regulatory experts—will be essential. By building a consensus on best practices, the field can transition from proof-of-concept experiments to robust, evidence-based guidelines that support effective biofilm control in medical and environmental settings alike.

## 5 Conclusion

The application of ultrasound in controlling biofilm adhesion presents a promising frontier for both research and clinical practice. Moving forward, investigations should focus on identifying optimal ultrasound frequencies and modulation techniques that maximize biofilm disruption while minimizing potential side effects. Combining ultrasound with advancements in nanotechnology and targeted drug delivery systems could further enhance its efficacy, offering more precise and effective treatment options.

The integration of real-time imaging and adaptive systems into ultrasound protocols may also refine treatment approaches, enabling interventions to be tailored to specific biofilm characteristics and their environmental contexts. Additionally, exploring combinatorial therapies that merge ultrasound with other physical or chemical modalities holds potential for improving outcomes in more challenging biofilm scenarios. While ultrasound has demonstrated significant promise, it is clear that overcoming its current limitations will require continued innovation. Its true potential lies in its adaptability and precision, as well as its capacity to synergize with complementary technologies. Future advancements will likely depend on a multidisciplinary approach that incorporates engineering, material science, and microbiology to address the complexities of biofilm control comprehensively.

Despite growing evidence supporting ultrasound-based strategies, the absence of standardized treatment protocols remains a barrier to widespread clinical adoption. Future efforts should aim to develop consensus guidelines that define optimal frequency, intensity, and duration tailored to specific biofilm and material contexts. Establishing such protocols will be critical to translating laboratory findings into routine clinical and industrial applications.
